# *Mycobacterium tuberculosis *complex drug resistance pattern and identification of species causing tuberculosis in the West and Centre regions of Cameroon

**DOI:** 10.1186/1471-2334-11-94

**Published:** 2011-04-15

**Authors:** Jean-Paul Assam-Assam, Veronique B Penlap, Fidelis Cho-Ngwa, Jean-Claude Tedom, Irene Ane-Anyangwe, Vincent P Titanji

**Affiliations:** 1 Biotechnology Unit, Faculty of Science, University of Buea, P.O. Box 63 Buea, Cameroon; 2 Laboratory of Tuberculosis Research, Biotechnology Centre of Nkolbisson, Faculty of Science, University of Yaoundé I, Yaounde, Cameroon

## Abstract

**Background:**

Data on the levels of resistance of *Mycobacterium tuberculosis *complex (MTBC) strains to first line anti-tuberculosis drugs in Cameroon, and on the species of MTBC circulating in the country are obsolete. The picture about 10 years after the last studies, and 6 years after the re-organisation of the National Tuberculosis (TB) Control Programme (NTBCP) is not known.

**Methods:**

The study was conducted from February to July 2009 in the West and Centre regions of Cameroon. A total of 756 suspected patients were studied. MTBC species were detected by the standard Ziehl-Neelsen staining method. Bacterial susceptibility to the first line drugs [isoniazid (INH), rifampicin (RIF), ethambutol (EMB) and streptomycin (SM)] were performed on cultures using the indirect proportion method. MTBC species were identified by standard biochemical and culture methods.

**Results:**

Of the 756 suspected patients, 154 (20.37%) were positive by smear microscopy. Of these, 20.77% were HIV patients. The growth of *Mycobacterium *was observed with the sputa from 149 (96.75%) subjects. All the isolates were identified as either *M. tuberculosis *or *M. africanum*. Among these, 16 (10.73%) were resistant to at least one drug (13.3% for the West region and 8.1% for the Centre). The initial resistance rates were 7.35% for the Centre region and 11.29% for the West region, while the acquired resistance rates were 16.66% (1/6) for the Centre region and 23.07% (3/13) for the West. Within the two regions, the highest total resistance to one drug was obtained with INH and SM (2.68% each). Multidrug-resistance (MDR) was observed only in the West region at a rate of 6.67%. No resistance was recorded for EMB.

**Conclusions:**

*M. tuberculosis *and *M. africanum *remain the MTBC species causing pulmonary TB in the West and Centre regions of Cameroon. Following the re-organisation of the NTBCP, resistance to all first line anti-TB drugs has declined significantly (*p *< 0.05 for West; and *p *< 0.01 for Centre) in comparison to previous studies. However, the general rates of anti-TB drug resistance remain high in the country, underscoring the need for greater enforcement of control strategies.

## Background

Every year approximately 9 million people contract tuberculosis (TB) and close to 2 million die from the disease [1]. The success of propagation of TB remains directly linked to the socio-economic and hygienic conditions of human populations [1]. While most TB cases are in Asia, the highest incidence rates are in Africa where high rates of HIV and malnutrition weaken immune systems and accelerate the spread of the disease [2]. Consequently, the number of new TB cases in most African countries has more than quadrupled since 1990, with 2.8 million new TB cases and roughly 735 thousand deaths annually [3]. The emergence of anti-tuberculosis drug resistance is a serious problem for TB control programmes in industrialized and developing countries alike. A global project on anti-tuberculosis drug resistance surveillance by the WHO and the International Union Against Tuberculosis and Lung Disease (IUATLD) [4] reported a prevalence of initial drug resistance of more than 10% in over 30 countries; it also identified 14 countries in which the prevalence of initial multidrug resistant TB strains (MDR-TB) (defined as resistance to at least isoniazid [INH] and rifampicin [RMP]) was more than 3%. In a 2000 WHO report on Africa, the overall level of initial resistance varied from 6.3% to 24.8%, and the level of MDR-TB from 1% to 5.3% [4]. However, the data available on the rates of drug resistance in Africa are not extensive and up to date.

In Cameroon, a country with over 18 million inhabitants distributed in 10 regions (previously provinces), the incidence of tuberculosis is relatively high, with more than 300 new cases per 100,000 inhabitants yearly [5]. Further contribution to the increased death rate due to TB in the country has been the emergence of drug-resistant strains to nearly all first line drugs [6]. RIF, INH, SM, and EMB are components of first-line multidrug therapy in Cameroon [7]. Between 1997 and 1998 in the West region of Cameroon, the overall resistance rate to at least one anti-TB drug was 26.9%, with initial resistance being 19.7% and acquired resistance 51.1% [6]. In the Centre region of the country, the rate of initial resistance to at least one drug in 1995 was 31.8%, and in 1998 it was 35.2% [8,9]. In 2000 the rate of acquired resistance was determined in the Centre region to be 58.2% [10]. Since then, no other report on the rates of resistance to these drugs in the country has been carried out. The rising prevalence of MDR strains has resulted in outbreaks and cases that are not only marginally treatable, but also often fatal. Following the awareness generated by the previous studies and the re-organsiation of the National TB Control Programme with a focus on proper treatment methods, it has been necessary to reassess the levels of resistance to the main anti-TB drugs in the country. Thus, in the present paper, we report on the current levels of resistance to these first line drugs in the West and Centre regions of Cameroon and identify the species of MTBC causing TB in the regions.

## Methods

### Study population and classification of samples

The study was conducted from February - July 2009 in six centres for the detection and treatment of TB (CDT) of the Centre region (Jamot Hospital, Mbalmayo District Hospital and Catholic Health Centre of Mvolye) and of the West region (Regional Hospital of Bafoussam, District Hospital of Baleng and District Hospital of Djeleng). Ethical permit was obtained from the Cameroon National Ethics Committee in Yaounde. In addition, each subject had to sign or thumbprint a patient consent form before they could be admitted into the study. A patient was confirmed sputum smear positive if at least two of three sputum samples collected over a 48 hour period were positive for acid fast bacilli (AFB). All sputum smear positive patients were included in the study. Patients confirmed as having pulmonary tuberculosis for the first time and without any history of previous anti-tuberculosis treatment were considered as new (or initial) cases and any drug resistance associated to them were reported as initial resistance. Patients with previous anti-tuberculosis treatment lasting a month or more were considered as old cases and any drug resistance based on them were reported as acquired resistance. Other pieces of information (notably age, occupation, gender, HIV status) were taken from the patients using a questionnaire. The sputum samples which were smear positive were kept temporarily at +4°C and transported within 48 hours to the *Mycobacterium *Laboratory of the Centre Pasteur du Cameroun in Yaoundé for bacteriological procedures.

### Bacteriological procedures

Each sputum sample was examined for acid-fast bacilli (AFB) by the standard Ziehl Neelsen staining method, and if positive, was cultured on Lőwenstein-Jensen (LJ) agar slants in tubes and in triplicate. One of the tubes was supplemented with sodium pyruvate at 0.4% to encourage the growth of any *M. bovis *present. The cultures were incubated at 37°C and examined weekly for a maximum duration of 10 weeks. Strain identification was based on the following criteria: growth rate, colony morphology, growth affinity for pyruvate, niacin production, reduction of nitrates and catalase activity [11].

Drug susceptibility testing was performed using the indirect proportion method on LJ medium as described by Canetti and collaborators [11]. The first line anti-tuberculosis drugs were tested as follows: rifampicin at 40 mg/l, isoniazid at 0.1 mg/l, isoniazid at 0.2 mg/l, streptomycin at 4 mg/l, and ethambutol at 2 mg/l. Drug resistance was defined as growth on a drug containing medium greater than or equal to 1% of that recorded on the drug-free control medium of the same experiment.

### Statistical analysis

Data were analyzed using Epi info 2000 version 12 or, when appropriate, by Fischer's Exact test, the Student`s t-test or the z-test for comparison of proportions. A difference was considered significant if *p *was < 0.05.

## Results

From a total of 756 subjects who presented clinical symptoms of tuberculosis at the hospital study sites, 154 (20.37%) were diagnosed positive by smear microscopy. They were 100 men (64.93%) and 54 women (35.07%) (Table 1). Patients' age distribution showed a steady decline in age-specific prevalence from 30.58% in age group 15-29 years to 15.51% in the age group above 60 years (Table 2). Of the 154 patients, 32 (20.77%) were HIV positive (Table 3); while 135 (87.66%) were new cases and 19 (12.33%) were old cases.

**Table 1:** Distribution of sputum samples collected

Region	Sites	Number of clinically suspected cases	Number of positive sputum smears
**Centre**	Jamot Hospital	75	39 (52%)
	District Hospital of Mbalmayo	125	27 (21.60%)
	Catholic health Centre of Mvolyé	113	9 (7.96%)
Subtotal		**313**	**75 **(**23.96%**)
**West**	District Hospital of Djeleng	130	35 (26.92%)
	Regional Hospital of Bafoussam	209	22 (10.52%)
	District Hospital of Baleng	104	22 (21.15%)
Subtotal		**443**	**79 **(**17.83%**)

**Total**		**756**	**154 **(**20.37%)**

**Table 2:** Age distribution of study subjects and patients

Age range (years)	15- 29	30-44	45 - 60	> 60
Number of suspected cases in age range (%)	206	216	122	**58**
Number positive by smear microscopy (% of positive cases)	63 (41.00%)	53 (34.41%)	29 (18.83%)	**9** **(5.84%)**

**% Smear positives in age group**	**30.58%**	**24.53%**	**23.77%**	**15.51%**

**Table 3:** Distribution of patients according to their HIV serological status

	*HIV Positive*	*HIV Negative*	Unknown HIV status
**Number of patients**	32 (20.77%)	103 (66.88%)	19 (12.33%)

The growth of *Mycobacterium tuberculosis *complex strains was observed with sputa from 149 (96.75%) of the 154 patients. The 5 samples without growth were thus excluded from further analysis. All the 149 isolates were identified as either *M. tuberculosis *or *M. africanum *species. Among these, 133 (89.26%) were susceptible to all the drugs tested, while 16 (10.73%) were resistant to at least one drug. This overall resistance rate was higher in the West region (13.3%) than in the Centre region (8.1%). Within the two regions, the highest resistance rate to one drug was obtained with INH and SM (2.68% each). We did not find any resistance to EMB in this study. Resistance to more than one drug stood at 4.69% (7 isolates) for the two regions (Table 4). A total of 4 isolates (2.68%) were resistant to two drugs only, and 3 (2.01%) to three drugs. We did not find any case of resistance to all four drugs (Table 4).

**Table 4:** First line anti-tuberculosis drug resistance pattern in the Centre and West regions of Cameroon

Resistance **to**:	Drug(s)	Region	Number of resistant cases in region	% in patients of region: N = 74 for Centre and 75 for West	Total % in patients of both regions
**One drug**	INH	Centre	2	2.70	2.68
		West	2	2.66	
	RIF	Centre	1	1.35	0.67
		West	0	0	
	SM	Centre	1	1.35	2.68
		West	3	4.00	
	EMB	Centre	0	0	0
		West	0	0	
**Two drugs**	INH, SM	Centre	2	2.70	1.34
		West	0	0	
	INH, RIF	Centre	0	0	1.34
		West	2	2.66	
**Three drugs**	INH, RIF, SM	Centre	0	0	2.01
		West	3	4.00	

**Totals to at least one drug**			16	8.10 (Centre); 13.30 (West)	10.73

(INH) = Isoniazid; (RIF) = Rifampicin; (SM) = Streptomycin; (EMB) = Ethambutol

The pattern of initial and acquired drug resistance is shown in Table 5. Initial resistance to at least one drug amongst the new cases of both regions (n = 130) was 9.23%, with resistance to INH being the most common (3.08%). The initial resistance rate was 7.35% (5/68) for the Centre region and 11.29% (7/62) for the West region. Acquired resistance to one drug amongst the old cases (n = 19) of both regions was 21.05%, with the resistance to SM being the most common (4/19 or 21.05%). The acquired resistance to at least one drug was 16.66% (1/6) for the Centre region and 23.07% (3/13) for the West region. Multidrug-resistant *M. tuberculosis *(MDR-TB) complex strains according to the definition were found in 5 (6.67%) of all the 75 isolates from the West region. By contrast, MDR-TB was not detected in any of the 74 isolates from the Centre region. MDR-TB was more frequently observed in the old cases than in the new cases (*p *< 0.05).

**Table 5:** Pattern of acquired and initial resistance to first line anti-tuberculosis drugs in the Centre and West regions of Cameroon

Resistance **to**:	Drug(s)	Region	Acquired resistance	% among old cases; N = 6 for Centre and 13 for West	Initial resistance	% among new cases; N = 68 for Centre and 62 for West
**One drug**	INH	Centre	0	/	2	**2.94**
		West	0	/	2	**3.22**
	
	RIF	Centre	0	/	1	**1.47**
		West	0	/	2	**3.22**
	
	SM	Centre	1	16.66	0	**0**
		West	0	/	1	**1.61**
	
	EMB	Centre	0	/	0	**0**
		West	0	/	0	**0**

**Two drugs**	INH, SM	Centre	0	/	2	**2.94**
		West	0	/	0	**0**
	
	INH, RIF	Centre	0	/	0	**0**
		West	0	/	2	**3.22**

**Three drugs**	INH, RIF, SM	Centre	0	/	0	**0**
		West	3	23.07	0	**0**

**Totals for both regions**			**4**	**21.05**	**12**	**9.20**

(INH) = Isoniazid; (RIF) = Rifampicin; (SM) = Streptomycin; (EMB) = Ethambutol

## Discussion and conclusions

Only a relatively small number of studies on anti-tuberculosis drug resistance have so far been carried out in Cameroon. The last of such studies carried out in the West (pilot region) and Centre (capital city being Yaounde) regions of the country were done over 10 years ago and before the re-organisation of the Cameroon National TB Control Programme [6,8,9]. The objective of the present study was to identify the mycobacterial species responsible for the spread of pulmonary TB in these two regions and to study the pattern of resistance of isolates to common first line anti-tuberculosis drugs in the regions. These regions are amongst the most socio-economically active in the country.

The growth of MTBC strains was observed with the sputa of 149 (96.75%) of the 756 suspected patients. The identification of the isolates by biochemical tests revealed the presence of only *M. tuberculosis *or *M. africanum *species. These results are in line with those of previous studies in the West and Centre regions of Cameroon where in 1995 and 1998, only these species were detected [8,9].

The majority of the patients were men (63.93%) and this was found to be in agreement with previous findings [12]. The prevalence of TB decreased steadily with age, with the youths being at greater risk of becoming active patients. The youths are socio-economically more active, and have the highest HIV infection rates in Cameroon. At least 32 (20.77%) of the 154 patients were HIV positive based on analysis of the questionnaires. The official prevalence of HIV infection in Cameroon is about 5.5%, indicating a greater than 4-fold increase in the prevalence of HIV infection amongst TB patients. On the global scale in 2007, 15% of all new cases of TB were infected with HIV [2]. Global figures suggest that HIV-positive people are between 6 to 37 times more likely than HIV-negative people to develop TB [2]. However, all the drug resistant MTBC isolates in the present study were from people who indicated they were HIV negative.

The overall resistance rates (one or more drugs) were 8.1% and 13.3% for the Centre and West regions respectively. The initial resistance rates were 7.35% and 11.29% for the Centre and West regions respectively, while the acquired resistance rates were 16.66% and 23.07% for the Centre and West regions respectively. Within the two regions, the highest resistance rates to one drug were obtained with INH and SM, while no resistance was recorded for EMB. A previous study carried out in the Centre region (capital city, Yaounde) of Cameroon in 1998 indicated an overall resistance rate of 35.2%, with initial resistance being 31.8% (164/516) and acquired resistance, 55.8% (48/86). In the study, resistance to streptomycin (20.5%), and isoniazid (12.4%) were the highest, while ethambutol showed the least (0.4%) [13,14]. A similar study carried out in the West region from 1997-1998 indicated the overall resistance rate to be 26.9%, with initial resistance being 19.7% (86/437) and acquired resistance, 51.1% (66/129). Again, the resistance to streptomycin (11.7%) and isoniazid (12.1%) were the highest, while the rates for ethambutol (2.5%) and rifampicin (2.1%) were low [6]. Our observations therefore follow the trends of these previous results obtained for the same regions, albeit a significant drop (*p *< 0.05 for West; and *p *< 0.01 for Centre) in resistance to all the drugs tested. This drop could be the fall-out of the reorganization of the National TB Control Programme, which emphasises the implementation of the directly observed treatment strategy (DOTs). The lower resistance rates for the Centre region in the present study could be accounted for, at least in part, by the fact that the region is the seat of the capital city of Cameroon, Yaounde where better health care facilities may be found. Both SM and INH have been in use in Cameroon for longer periods than RIF and EMB. However, a compilation of 63 surveys on resistance to anti-tuberculosis drugs carried out between 1985 and 1994 in some countries showed the rate of acquired EMB resistance to be as high as 13.7% [15]. The rate of initial resistance in the present study was found to be higher (9.2% for the two regions) than that reported from other African countries such as Zimbabwe (3.3%) and Bostwana (3.7%) [16], but lower than those reported in similar studies from Uganda [17], Ivory Coast [16], and Sierra Leone [16] (27.4%, 13.4%, and 28.1% respectively). In some African countries the rates of acquired drug resistance are reported to be lower or similar (e.g. Zimbabwe, 13.9%; Botswana, 14.9%; and Swaziland, 20.5%) [16].

A more serious aspect of the TB drug problem is when the infecting organism is resistant to both INH and RIF, referred to as multi-drug resistant TB (MDR-TB) [18]. Under this condition, the duration of treatment is prolonged from 6 to 18-24 months, and the cure rate could decrease from nearly 100% to less than 60%. This makes the treatment of multi-drug-resistant cases particularly challenging [19]. MDR-TB strains were found in 5 (6.67%) of the 75 isolates from the West region. No MDR-TB strain was detected in any of the 74 isolates from the Centre region. A similar work done in the West region in 2000 indicated an MDR-TB rate of 4.1% [6]. This indicates a slight increase in the rate of MDR-TB. MDR-TB is an important problem globally [20]. It results from deficiencies in TB case management and control programmes, and there is growing evidence now of an overlap between this epidemic and HIV infection [21]. In 2004, it was estimated that 4.3% of all new and previously treated tuberculosis cases world-wide were multidrug resistant [22]. The results of a worldwide survey showed that in 2006, 2% of *M. tuberculosis *isolates were extensively multidrug resistant (XDR-TB, caused by MDR strains also resistant to quinolones plus one of the injectable second line agents) [23].

We conclude that *M. tuberculosis *and *M. africanum *remain the MTBC species causing pulmonary TB in the West and Centre regions of Cameroon, and that following the re-organisation of the NTBCP, resistance to all first line anti-TB drugs has declined significantly in comparison to previous studies, with ethambutol now showing no resistance in both regions. However, the general rates of anti-TB drug resistance remain high in both regions, underscoring the need for greater enforcement of control strategies in the country.

## Competing interests

The authors declare that they have no competing interests.

## Authors' contributions

JPAA carried out many of the experiments as a PhD student, participated in field work and drafted the manuscript; VBP participated in field work and in the conception, design, supervision of the experiments, analysis of data and revision of the manuscript; FCN participated in field and laboratory work and in the conception, design, supervision of the experiments, analysis of data as well as in the drafting and revision of the manuscript; JCT assisted JPAA in the field and on the laboratory bench; IAA participated in the field work and in the revision of the manuscript; VPT was the overall supervisor and chief designer of the project. All authors read and approved the final manuscript before submission.

## Pre-publication history

The pre-publication history for this paper can be accessed here:

http://www.biomedcentral.com/1471-2334/11/94/prepub
